# Careless Writing

**Published:** 1862-07

**Authors:** 


### Editorial.

### CARELESS WRITING.

A CONVERSATION took place at a jail window between a London porter, a soldier on guard duty, and a prisoner for life inside. The subject was a certain act of Parliament. "It will deprive us of our liberty," said the prisoner. "A fatal stab at our holy religion," solemnly ejaculated the soldier. "And will force us to carry burdens for a living," groaned the other from under his load. Thus each was distressed in a direction in which he, at least, was not very likely to be worsted.

We were reminded of this by something else:

In a certain periodical, "devoted to the interests of the profession," two prominent writers, moved by kindred impulses, tell us —the one how to write "articles for the periodicals"—the other how to *use* and avoid the *abuse* of words. In doing so, the former perpetrates the most carelessly written article we have ever seen in any periodical; and the latter abuses words without measure. The former hopes that writers will not "write long, labored, mystical, and altogether theoretical and lumbering articles for the journals," while he is rioting in such sentences as this:—"It is not reasonable that a fistulous opening can be healed up of a deciduous tooth as readily and as permanently as an adult tooth, when its fang is completely formed or ceased growing, the changing character and the increased vascularity consequent upon absorption of the deciduous roots favors the persistence of the fistulous openings in them!"

Now, this sentence is certainly "mystical." Can an adult tooth be healed at all, even temporarily? And if not, far less can it be healed "readily" and "permanently." The sentence, too, is "labored." When words are allowed to come of their own accord, they never place themselves in such ludicrous arrangements. Nothing but great labor can so distort a sentence. It is "lumbering," too. It moves heavily. It lacks but little of being "altogether theoretical;" and it is certainly "long."

But, if it is thought that this is not a fair specimen, take another sentence:—"The healing is favored more by keeping the root of a tooth open, except cotton and creosote and a tent of cotton in the fistulus in the gum, than medicaments; consequently, they are of little account in children," That is *mystical*, too, especially the "fistulus," which the reader not posted in Latin will understand is either a print-error, or a *he fistula*.

The other writer, though guilty of nothing so cruel as the above, has no mercy on grammar. For example:—"Who has not noticed how differently the same thing, said in different ways, affect them!"

Now, although the last verb has a strong *affection* for the "thing" in the former part of the sentence, he unmercifully kicks its *s* out of company, and sends it in search of "ways" that it knows not.

And, with like carelessness, we have arsenious acid, when applied to the foot of a healthy frog, called a "remedy."

We have thus alluded to errors which are by no means lonely in the compositions of two of the most prominent dental writers.

We know that the matter does not concern us personally, but professionally it does. It concerns the humblest member of our profession. Writers are judged by their styles. And if such are the high priests, what are the people? Men of talent have no right to indulge in such carelessness; for it is nothing else in the cases referred to. If not sufficiently compensated, they should either cease their labor, or perform it aright for its own sake.

We expect our motive in writing this to be misunderstood, as usual, in a certain quarter. W.

